# Venereal Cases in General Institutions. A Public Danger?

**Published:** 1919-03-08

**Authors:** Gladys M. E. Leigh


*To the Editor of* THE HOSPITAL.

### Venereal Cases in General Institutions. A Public Danger?

*To the Editor of* THE HOSPITAL.

SIR,—We read daily concerning the measures taken for the stamping out of venereal disease and the prevention of the spread of infection.

We are told the improvement of the national health is a major consideration amongst civilised communities, yet patients suffering from specific disease are admitted into West-end Homes. Moreover, the nurses attending these cases are allowed to nurse ordinary surgical patients, and no special precautions are observed. If the public were generally aware of these facts surely there would be raised such a unanimous protest as would force the authorities to take action rendering this state of affairs impossible.—Yours faithfully, GLADYS M. E. LEIGH.

